# Prediction of dyskinesia in Parkinson’s disease patients using machine learning algorithms

**DOI:** 10.1038/s41598-023-49617-w

**Published:** 2023-12-16

**Authors:** Denisson Augusto Bastos Leal, Carla Michele Vieira Dias, Rodrigo Pereira Ramos, Ivani Brys

**Affiliations:** 1 Postgraduate Program in Health and Biological Sciences, Federal University of Vale do São Francisco (UNIVASF), Av José Sá de Maniçoba s/n, Petrolina, 56304-917 Brazil; 2 Postgraduate Program in Psychology, Federal University of Vale do São Francisco (UNIVASF), Petrolina, Brazil

**Keywords:** Parkinson's disease, Prognostic markers, Scientific data, Computational science

## Abstract

Dyskinesias are non preventable abnormal involuntary movements that represent the main challenge of the long term treatment of Parkinson’s disease (PD) with the gold standard dopamine precursor levodopa. Applying machine learning techniques on the data extracted from the Parkinson’s Progression Marker Initiative (PPMI, Michael J. Fox Foundation), this study was aimed to identify PD patients who are at high risk of developing dyskinesias. Data regarding clinical, behavioral and neurological features from 697 PD patients were included in our study. Our results show that the Random Forest was the classifier with the best and most consistent performance, reaching an area under the receiver operating characteristic (ROC) curve of up to 91.8% with only seven features. Information regarding the severity of the symptoms, the semantic verbal fluency, and the levodopa treatment were the most important for the prediction, and were further used to create a Decision Tree, whose rules may guide the pharmacological management of PD symptoms. Our results contribute to the identification of PD patients who are prone to develop dyskinesia, and may be considered in future clinical trials aiming at developing new therapeutic approaches for PD.

## Introduction

Parkinson's disease (PD) is the second most common neurodegenerative disorder after Alzheimer's disease, affecting 1% of the world population over the age of 60 years, and 3% over the age of 80 years^1^. According to^2^, it is estimated that currently 10 million people in the world live with PD. The United States has approximately a million PD patients and by 2030 it is expected to reach 1.2 million.

Currently there is no cure for PD, and the gold standard treatment aims to relieve the PD symptoms through the drug levodopa, which is a dopaminergic precursor^3^. Levodopa is effective in relieving PD symptoms in the short term, but in the long term it is associated with the development of motor complications known as dyskinesias, which compromise the pharmacological management of parkinsonian symptoms and affect the life of the patients. After five years of levodopa treatment, it is estimated that 50% of patients develop dyskinesia, and after ten years, 80%^4,5^.

Recent studies have shown that the levodopa dosage, the female gender, high levels of anxiety and severe motor impairments are risk factors for the development of dyskinesias^6–8^. However, dyskinesias are considered not preventable and there is no differentiation between patients who are prone to develop dyskinesia and those who are not in the clinical practice or in clinical trials aiming at developing new treatments for PD.

While hard to detect in the clinical and individual practice by health professionals, predictors of dyskinesia might be identified by machine learning techniques in vast amounts of data from PD patients. Machine learning algorithms have already been used for similar purposes, such as improving PD diagnosis^9,10^, and quantifying the severity of PD symptoms based on smartphone assessments^11^.

The Parkinson’s Progression Marker Initiative (PPMI) is a longitudinal study conducted by the Michael J. Fox Foundation aimed to identify PD biomarkers and provide therapeutic trials with collaborative tools^12^. In this study, hundreds of PD patients were followed for several years during which neurological and clinical assessments, imaging examinations, and biological sample collections were systematically performed. The PPMI database represents therefore a unique set of data regarding the longitudinal follow-up of PD patients, through which it is possible to identify in a universe of several features, markers that precede the onset of dyskinesias in patients undergoing levodopa treatment.

Applying machine learning techniques to the PPMI dataset, the objective of this study was to identify PD patients who are at high risk of developing dyskinesia and the most important features for this prediction. We further created a decision tree aimed at providing explainability to our method, and show how each feature influenced the classification. The rules of this decision tree may be applied to differentiate patients in future clinical trials aiming at developing new therapeutic or preventive approaches for dyskinesia, and may also help to guide the dopaminergic therapy of PD in the clinical practice.

## Patients and methods

**Figure 1:**
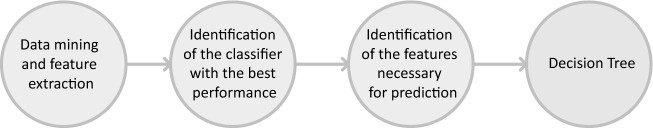
Sequence of the steps performed in the project. Step one—Data mining and feature extraction: we first identified 53 clinical and behavioral features regarding motor, non motor and neurological characteristics of the patients in the PPMI dataset. For each of these features, we identified the score of each patient in the assessment that preceded the onset of dyskinesia onset or in the latest assessment available for the non dyskinesia group. Step two—Identification of the classifier with the best performance: We compared the performance of seven different classifiers identifying the patients that were prone to develop dyskinesia. The classifier with the best performance and the lowest variability was the one used in the further steps. Step three—Identification of the features necessary for prediction: A minimum quantity of features was then identified as necessary and sufficient for the classification. Step four: Decision tree: a decision tree was created using only the features regarded as sufficient for classification.

This study was conducted in four steps, as illustrated in the flowchart shown in Fig. 1. The first step consisted of extracting characteristics of PD patients from the PPMI database; the second consisted of assessing the performance of seven classifiers to identify which one would have the best performance classifying the patients with and without dyskinesia using all the 53 features extracted in step one. In the third step, we tested the performance of the classifier found in step two, with the objective of finding the minimum quantity of features necessary for prediction. This step is important considering a clinical scenario where collecting information on more than 50 variables from patients would be unfeasible. Finally in the fourth and last step, we chose the operating point of the classifier and assembled some rules to use the features found in step three with the help of a decision tree. The rules of this decision tree provide information on how each variable influenced the classification, and help to translate the classification into terms of clinical assessments. The steps are described in detail below.

**Compliance with ethical standards:** The present study was performed with data from non-identified participants and, therefore, was not submitted to the appreciation of any local Ethical Committee (Resolution 510/2016 of the National Health Council, article 1, chapter V). The PPMI dataset is publicly available and the original study was performed in accordance with the Declaration of Helsinki. Each PPMI center received approval from the respective ethics committee before starting the study. Written consent for research was obtained from all participants, as described in the PPMI study protocol, available in https://www.ppmi-info.org/study-design/research-documents-and-sops/.

### PPMI dataset and data extraction

Data used in the preparation of this article were obtained from the Parkinson’s Progression Markers Initiative (PPMI) database (www.ppmi-info.org/access-data-specimens/download-data, RRID:SCR_006431. For up-to-date information on the study, visit www.ppmi-info.org).

The PPMI database (Michael J. Fox Foundation) contains longitudinal information of PD patients with no genetic mutation or with one of the following mutations: LRRK2, GBA or SNCA. The data consist of genetic, socio-demographic, behavioral, and neurological information collected through laboratory tests, imaging tests, application of scales and questionnaires that were filled out by the patient, caregiver or a family member. For the present study, the dataset was accessed on October 11, 2020, when data from 795 PD patients distributed in 141 worksheets were downloaded. The main inclusion criteria for PD subjects participating in the PPMI study were: presence of at least two cardinal symptoms of PD, or either asymmetric resting tremor or asymmetric bradykinesia; diagnosis of PD for two years or less; and dopamine transporter deficit confirmed by imaging screening.

Given that our objective was to predict dyskinesia, only patients that completed at least one assessment before the onset of dyskinesia were included in our study. Patients were considered dyskinetic when they scored ≥ 1 in the item “Time spent with dyskinesias” of the Movement Disorder Society Modified Parkinson's Disease Rating Scale. Patients with no dyskinesia and taking amantine were excluded due to the antidyskinetic effects of this drug^13^. Patients with more than 15% of missing data were also excluded.

**Figure 2:**
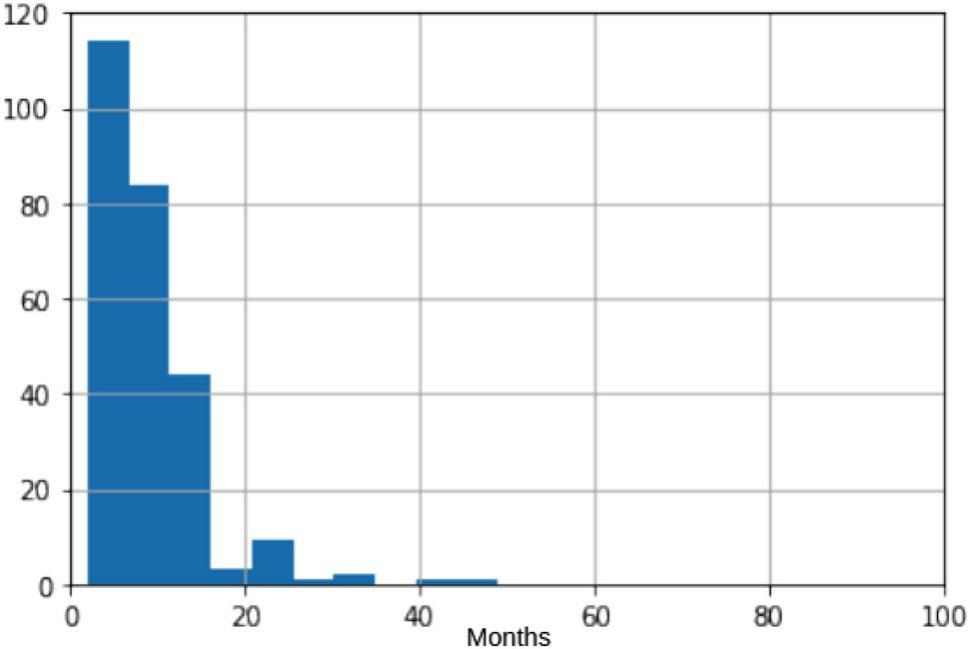
Histogram showing the time interval between the patient's assessment and the onset of dyskinesia for patients in the PD with dyskinesia group.

As a result of these inclusion and exclusion criteria, data from 697 patients were included in our study. In order to maximize the number of patients in our study, we used the data of the last visit preceding the onset of dyskinesia for each patient. The mean time interval between the data collection and the onset of dyskinesia was 9 months with a standard deviation of 6.1 months. Figure 2 shows the distribution of the time interval between the patient assessment and the onset of dyskinesia in the group of patients with dyskinesia. For patients with no dyskinesia, data from the latest available assessment was used. In our dataset, the data granularity, defined as the size in which data fields are subdivided, consisted of motor, non-motor, and neurological features of each patient (Table 1).

**Table 1:** Clinical, motor, non-motor, and neurological features used in the project with the names and acronyms used.

Features
Age
Education
Age of PD diagnosis
PD disease duration
Family history of PD
Man
Woman
Movement Disorder Society Unified Parkinson Disease Rating Scale (UPDRS)—part I Patient Questionnaire (PQ)
UPDRS—part I total
UPDRS—part II
UPDRS—part III
UPDRS—part III ON levodopa (A)
UPDRS total
levodopa Equivalent Daily Dose (LEDD) levodopa
Levodopa LEDD time—duration of levodopa treatment
maob inhibitors LEDD
maob inhibitors LEDD time
dopamine agonists LEDD
dopamine agonists LEDD time
comt inhibitors LEDD
comt inhibitors LEDD time
Amantadine LEDD
Amantadine LEDD time
Total LEDD
LEDD time—duration of dopaminergic therapy
Mean Putamen
Asymmetry Putamen
Contralateral Putamen
Mean Caudate
Asymmetry Caudate
Contralateral Caudate
Alpha synuclein (cerebrospinal fluid values)
Semantic Verbal Fluency test (SVF)
Symbol Digit Modalities Test (SDMT)
Hopkins Verbal Learning Test (HVLT)
Physical Activity Scale for the Elderly (PASE)
State-Trait Anxiety Inventory (STAI)
State Anxiety Inventory (STAI—I)
Trait Anxiety Inventory (STAI—II)
Scales for Outcomes in Parkinson's Disease (SCOPA-AUT)
Epworth Sleepiness Scale (ESS)
Tremor dominant (TD) score
Postural instability and gait disturbance (PIGD) score
PIGD vs TD scores
Number sequencing (*Wechsler Memory Scale*—WMS-III66)
REM Sleep Disorder Questionnaire (RBDSQ)
Benton Judgment of Line Orientation Test (BJLOT)
University of Pennsylvania Smell Identification Test (UPSIT)
Montreal Cognitive Assessment (MOCA)
Modified schwab and england activities of daily living scale (ADL)
Geriatric Depression Scale (GDS)
Questionnaire for Impulsive-Compulsive Disorders in Parkinson’s Disease Current Short (QUIP)

The data summary and the number of patients in each class with and without dyskinesia are shown in Table 2. Note that the proportion of patients with dyskinesia in the subgroups genetic mutation, gender or with a family history of PD does not vary more than five percentage points in each class, and thus the patients were not distributed in subgroups for further analysis.

**Table 2:** Proportion of patients with and without dyskinesia, distributed according to the presence of a genetic mutation, gender and the family history of PD.

	Dyskinesia		No dyskinesia		Total
Patients in the study	238	34.1%	459	65.9%	697
With genetic mutation	84	30.3%	193	69.7%	277
Male gender	143	34.7%	269	65.3%	412
Female gender	95	33.8%	186	66.2%	281
Family history of PD	83	31.2%	183	68.8%	266

### Preprocessing

For some features, the absence of a value represents that it has no such value and can therefore be filled out with zero. This is the case of the variable LEDD, whose missing values indicate that the patient was not taking medication with dopaminergic action. For gender, a similar rule was applied. When the patient did not have a value indicating the gender, no technique was used to replace the missing value. In all other cases the missing values were identified and filled out using the non parametric imputation method MissForest (829 in total), which employs the Random Forest algorithm to make predictions using the existing records in an automatic and personalized way for each case^14^. The few categorical data were organized in columns using the standard one-hot encoding, and the continuous data were normalized between 0 to 1 using the MinMax standard.

The dataset was then randomly subject-wise split into training (60%), validation (20%), and test (20%), without repetition and keeping the same proportion of classes with and without dyskinesia in each subdivision.

### Classifiers

At this stage we used three simple classifiers: decision tree, multinomial bayesian and logistic regression), two classical classifiers: Multilayer Perceptron (MLP) and Support Vector Machines for classification (SVC), and classifiers with ensemble: Adaboost and Random Forest. Before training the classifiers, a search for the best combination of hyperparameters was performed using the grid search, with all possible combinations allowed by the library, except in cases where the combination would be infinite, such as the number of layers and neurons of the MLP or the number and depth of the Random Forest trees (Supplementary material 1). The Area Under the Curve (AUC) Receiver Operating Characteristics (ROC) was calculated to identify the classifier with the best performance. In order to ensure a fair performance presentation of the classifiers, each one was trained and tested 30 times, with training and validation data randomly changing over turns. The results were used to construct a boxplot with the ROC AUC in order to observe the variability of the results. The accuracy, the ROC curve, the true and false negative rate were then calculated for the classifier with the best performance.

The ROC curve was used to define an operating point for the classifier with the best performance. The operating point is a value that divides the results between classes that, in our case, corresponded to patients with or without dyskinesia. This point may be empirically defined with the help of an expert or according to the performance of the classifier. In the present study, several operating points were compared in order to find one where the largest number of dyskinetic patients were accurately classified. First, we calculated the default operating point, using a threshold of 0.5. Next, we calculated the Youden index using the largest distance between the ROC curve and the random choice line. Finally, only points with a true positive rate of at least 95% or 100% were selected.

### Decision tree

**Figure 3:**
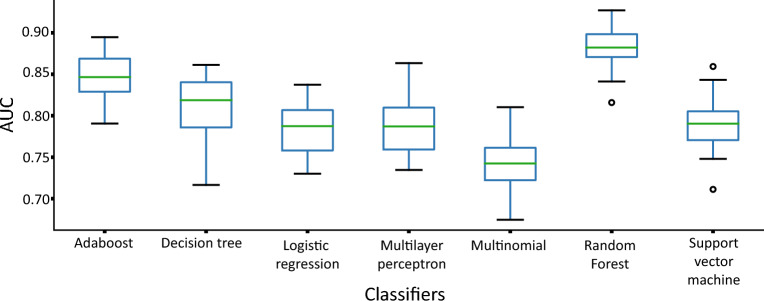
Random Forest was the classifier with the best performance and the lowest variability. Boxplots represent the lower limit with the minimum value, first quartile with 25% of the data, the median with 50% of the data, the third quartile with 75% and finally the upper limit with the maximum value. The circles represent outliers.

The decision tree uses a tree structure, similar to a binary tree, where an algorithm is used in each node to make a decision on how the data may be divided. At the end of the decisions (in each leaf of the tree) the final decision on the classification or regression of the target variable is made^15^. Unlike the Decision tree shown in Fig. 3 that was created with the purpose of achieving the best performance in classification using all the features extracted from the PPMI database, this Decision tree aims to be a visual tool and provide an overview of the data for rules definition, using only the features regarded as necessary for classification in the previous step.

For this purpose, the Classification and Regression Trees (CART) algorithm was used to create a decision tree and its rules using the whole dataset. The gini index was computed to measure the quality of the nodes division, and in order to prevent rules from becoming too specific to the problem^16^, a limitation was imposed to not generate nodes with less than 10% of the cases.

For each node, the coverage, the confidence and the lift were calculated to assess the quality of the rules. The coverage refers to the percentage of entries that passed through a given node. The confidence refers to the percentage of data arriving at a given node that belongs to the target class i.e. with dyskinesia. Finally, the lift is the ratio between the node confidence and the overall confidence. Thus, a lift score different from one indicates that the rule effectively separates the data^17^.

## Results

The first step of the present study was aimed to identify the classifier with the best performance classifying the patients using all the features extracted from the PPMI dataset. In this experiment, the classifiers Adaboost, Decision Tree, Logistic Regression, MLP, Multinomial, Random Forest, and SVM were run 30 times (bootstrap), using all the 53 features described in Table 1 as data source. Figure 3 shows the boxplots with the AUC ROC for each classifier.

Through a visual inspection of Fig. 3, it is possible to notice that the upper limit, the lower limit, the quartiles and the median of the Random Forest classifier were higher compared to the others and that its performance presented less variability among turns. Therefore, the Random Forest was the classifier used in the further steps of our study.

**Figure 4:**
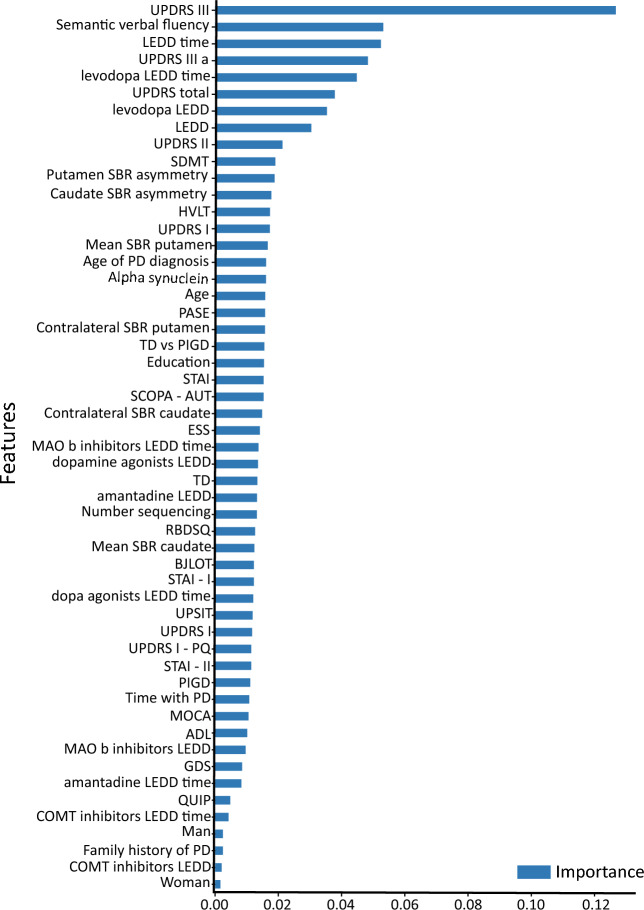
Features importance according to the Random Forest classifier. Bars represent the importance given by the classifier, i.e., the higher the bar, the more important the feature.

Next, we ranked all the features according to their importance for the classifier, as shown in Fig. 4. The higher the score assigned to the feature, the greater the importance it had for the classifier. Part III of the UPDRS was the feature with the highest importance, indicating that for the Random Forest classifier the severity of PD motor symptoms was the most important feature to differentiate patients who are about to develop dyskinesia from those who are not.

**Figure 5:**
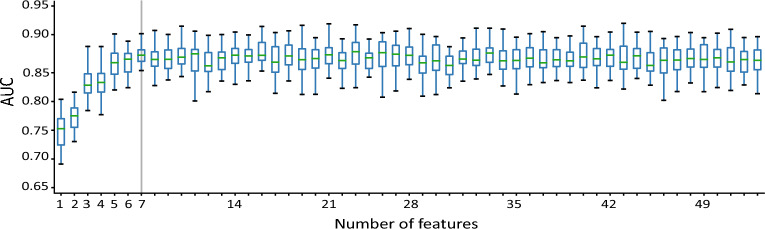
Random Forest classification performance varies when increasing the number of features. From left to right, starting with one feature and adding up to all 53 features, the boxplots show the performance of the classifier over 30 turns. The gray line shows when the performance becomes stable.

Considering the potential applications of our results in a clinical context, we used the order of importance of the features to investigate what is the minimum number of features needed for a good prediction using our classifier. The Random Forest classifier was then trained and tested for each number of features used from the most important to the least, starting with only one feature adding up to all 53 features. Such as in the previous experiments, the Random Forest classifier was run 30 times and the resulting boxplots are shown in Fig. 5.

Figure 5 shows that the Random Forest is able to classify the PPMI patients using only seven of the 53 features tested, with an AUC ROC varying from 85.4 to 91.8%, and median equal to 88.1%. Adding more features to the classifier did not increase the classification performance and may therefore be considered unnecessary to the present problem.

**Figure 6:**
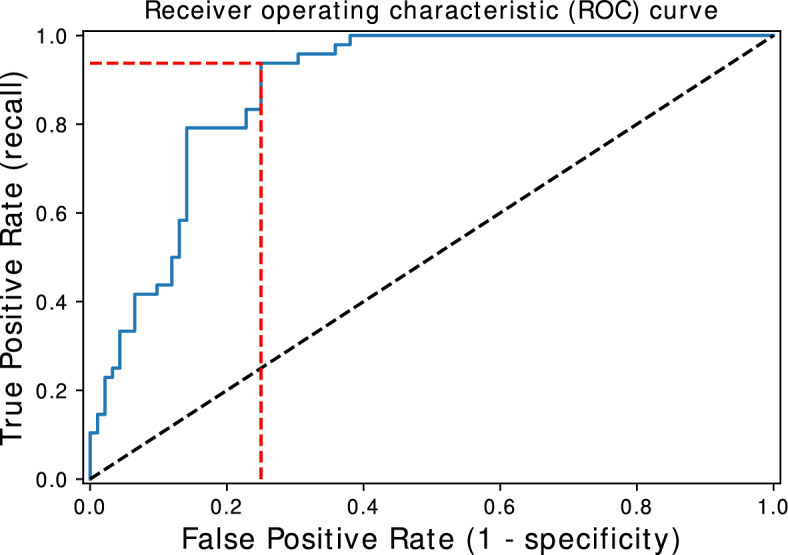
ROC curve for the median performance of the Random Forest using the seven features identified as necessary for classification. The intersection of the red dashed lines corresponds to the Youden index. The black dashed straight line shows the result of a random classifier, used for comparison. The blue curve shows the true and false positive rates when varying the threshold; the steeper the curve, the better the classifier.

The seven necessary features for the classifier were: the UPDRS III score, the Semantic Verbal Fluency test score, the duration of the dopaminergic treatment, the UPDRS III part A score i.e. ON levodopa, the duration of the levodopa treatment, the total UPDRS score, and the levodopa LEDD. In order to design a more realistic scenario considering the clinical context, we plotted in Fig. 6 the ROC curve for the Random Forest median performance, with the ROC AUC of 88.1%, using only the seven features regarded as necessary and sufficient for the classification. We further assessed some operation points considering that, for a potential preventive strategy, it would be preferred to identify the highest possible number of patients at risk of developing dyskinesia, even if it leads to the inclusion of some patients who are not at risk, that is, resulting in more false positives. Starting with the most common operation point, threshold of 0.5, the classifier accuracy decreased to 80.7% with a true positive rate of 70.8%, slightly below the point chosen by the Youden index, which resulted in an accuracy of 81.4% and a 93.8% true positive rate. When we prioritized an operation point where the true positive rate was 95% or 100%, the accuracy dropped to 78.6% and 75%, and the false positive rate rose to 30.4% and 38%, respectively. Thus, we concluded that the operating point found by the Youden index showed the best balance between accuracy and the true positive rate.

The operation point of the Youden index was obtained with a threshold around 0.3334, which means that when the classifier says that a record has a probability greater than or equal to this value, it is classified as having dyskinesia. Table 3 shows the confusion matrix generated with this classification. It is important to note that these results were obtained for only 34.1% of the PPMI patients who showed dyskinesia. According to the literature this proportion is expected to increase up to 50% after five years of levodopa treatment, and up to 80% after ten years^4^. Considering this scenario and if the rate of true positives remains around 93.8%, it is likely that the accuracy increases with time.

**Table 3:** Confusion matrix generated using the Youden index.

		predicted	
		Non dyskinesia	dyskinesia
current	Non dyskinesia	69	23
	dyskinesia	3	45

True negatives are the values without dyskinesia correctly classified. True positives are those with dyskinesia correctly classified. False negatives are patients with dyskinesia misclassified as without dyskinesia and false positives are the patients who do not have dyskinesia, and were misclassified as having dyskinesia.

**Figure 7:**
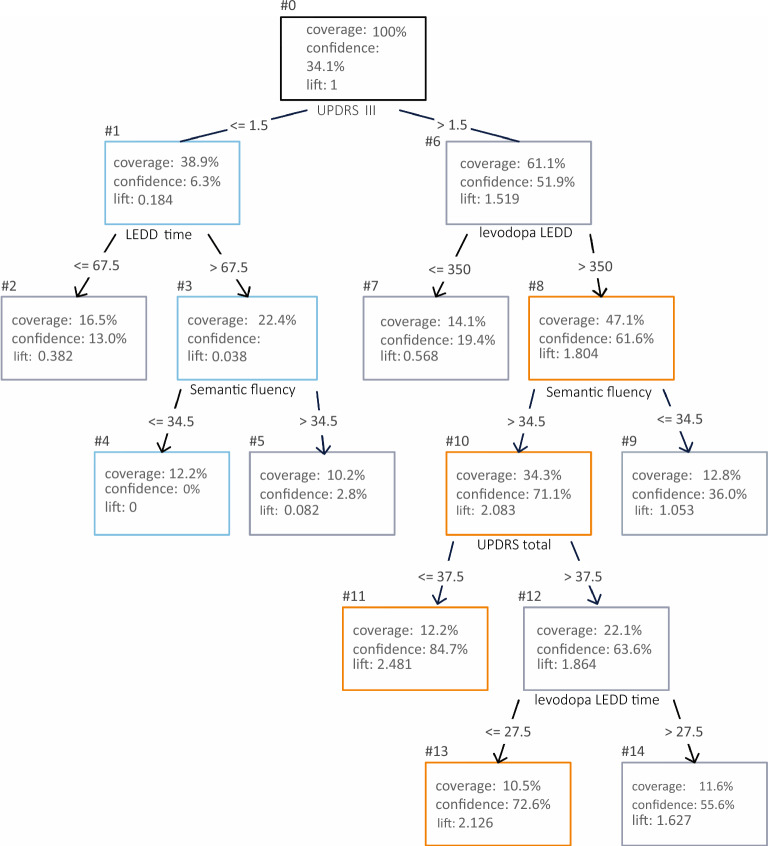
Decision tree using the seven features necessary for classification. Orange color indicates rules that are good to identify patients that would develop dyskinesia, and light blue indicates rules that are good to identify patients that would not. The paths from the root to any node correspond to the rules and do not have to necessarily reach any leaf. The nodes of the tree have a minimum coverage of 10% and the more distant from 1 the lift is the better the rule.

The last step of our study was aimed to find the main classification rules using a decision tree created with the CART algorithm. The training step was performed with the seven features found in the previous experiments and with all patients in the dataset. The assembled tree is illustrated in Fig. 7, where the coverage, confidence and lift in each node, and the condition applied for the division of the subsequent nodes are shown.

Considering the results of the Decision Tree, the rules 1, 3 and 4, shown in Table 4 had low confidence in relation to the target class, which means that they are good to identify patients without dyskinesia. For example, approximately 12% of the data matched rule 4 and none of them are part of the target class i.e., none has dyskinesia. This does not mean that none, in the entire universe of patients, will have dyskinesia. However, considering the representativeness of the PPMI dataset, the chance is very low. The rules 3 and 1, on the other hand, had higher coverage, 22.4% and 38.9% and low confidence, 1.3% and 6.3% respectively, which means that they are good framing rules for patients without dyskinesia.

**Table 4:** Decision tree rules that identified mainly patients with low risk of developing dyskinesia.

	Rule	Coverage (%)	Confidence (%)	Lift
4	UPDRS III score lower or equal to 1.5, and LEDD time larger than 67.5 months, and semantic fluency index lower or equal to 34.5	12.2	0.0	0.000
3	UPDRS III score lower or equal to 1.5, and LEDD time larger than 67.5 months	22.4	1.3	0.038
1	UPDRS III score lower or equal to 1.5	38.9	6.3	0.184

Rule 5 also showed low confidence, and it is very similar to rule 3, with a slightly higher confidence and a slight decrease in coverage. Thus, it is expected that all cases of rule 5 are also in rule 3, which showed to be a better rule. According to our results, patients who fit rule 4, i.e., who have UPDRS III score lower or equal to 1.5, LEDD time larger than 67.5 months, and semantic fluency index lower or equal to 34.5, have a very low chance of developing dyskinesia. These rules may be used to differentiate PD patients with low risk of developing dyskinesia in future clinical studies and in the clinical practice.

On the other hand, rules 8, 10, 11 and 13 shown in Table 5, differentiated mainly patients that were about to develop dyskinesia. Rules 13 and 11 had lower coverage, slightly higher than 10%, but both had high confidence of the target class, 72.6% and 84.7%, respectively, higher than the overall confidence of 34.1%. Rules 8 and 10 showed higher coverage but lower confidence levels which are still almost twice the overall confidence, meaning that patients who fulfill these rules are almost twice as likely to develop dyskinesia. Rule 12 is another example of a rule that, even with a high lift, ended up within the rule 10 that had higher coverage and lift.

**Table 5:** Decision tree rules that identified mainly patients with high risk of developing dyskinesia.

	Rule	Coverage (%)	Confidence (%)	Lift
8	UPDRS III score higher than 1.5, and levodopa LEDD greater than 350	47.1	61.6	1.804
10	UPDRS III score higher than 1.5, and levodopa LEDD greater than 350, and semantic fluency index higher than 34.5	34.2	71.1	2.083
13	UPDRS III score higher than 1.5, and levodopa LEDD greater than 350, and semantic fluency index higher than 34.5, and UPDRS total score higher than 37.5, and levodopa treatment duration shorter than 27.5 months	10.5	72.6	2.126
11	UPDRS III score higher than 1.5, and levodopa LEDD greater than 350, and semantic fluency index higher than 34.5, and UPDRS total score lower or equal to 37.5	12.2	84.7	2.481

In the inverse of the low confidence rules, the rule 11 accurately identified 84.7% of patients with UPDRS III score higher than 1.5, levodopa LEDD higher than 350, semantic fluency higher than 34.5 and the total UPDRS score lower than or equal to 37.5 as dyskinetic. Although presenting lower confidence, rules 8, 10 and 13, had similar characteristics, and may also be used in preclinical studies or in a clinical setting to differentiate the PD patients that are at high risk of developing dyskinesia.

A more detailed look at the rules of the decision tree revealed that the semantic fluency generated good rules in both nodes where it appeared. In both cases a semantic fluency lower than 34.5 led to rules with low lifts. The condition where the UPDRS III score is lower or equal to 1.5 is also a quite interesting case. All rules with this condition had lift values below 1, showing that this feature was able to adequately divide the data. Levodopa LEDD values lower than or equal to 350 mg also resulted in rules with the lift values below one, even when the patient had the UPDRS III score greater than 1.5.

## Discussion

In this study, we applied several machine learning techniques to the PPMI database in order to identify the most important clinical, behavioral and neurological features for the prediction of dyskinesia in PD patients. The Random Forest classifier had the most accurate and consistent performance with an AUC ROC of up to 91.8% and a median of 88.1% using only seven features.

Our results showed that the score of the patients on part III of the UPDRS had the highest importance for the classification, indicating that the severity of PD motor symptoms was the most important feature for the Random Forest classifier when differentiating patients who are about to develop dyskinesia from those who are not. These findings are in accordance with the literature showing that dyskinesia appears as a result of the interaction between chronic dopaminergic therapy and the progression of striatal dopamine denervation^18^.

Following the UPDRS III score, other six features showed to be important for dyskinesia prediction in our analyses: the Semantic Verbal Fluency test score, the duration of the dopaminergic treatment, the UPDRS III part A score i.e. ON levodopa, the duration of the levodopa treatment, the total UPDRS score, and the levodopa LEDD. These findings are partially in accordance with the results of Nicoletti et al.^19^ showing that the disease duration, the Hoehn-Yahr stage, the UPDRS score, the female gender and the duration of the dopaminergic therapy are associated with the development of dyskinesias. Similar results were presented by Eusebi et al. (2018), who also used a Random Survival Forests classifier to determine the risk factors for the development of dyskinesias in PD patients. Compared to our findings, the main differences concern the inclusion of the genetic risk variable in that study, and the importance of gender. While for Eusebi et al.^6^ and Nicoletti et al.^19^ being female was considered a risk factor for dyskinesia, in our study, the gender of the patient was one of the least important features. These differences may be explained by updates in the database itself, given that our most recent access happened in 2020, 2 years after the publication of the cited article. In addition, differences in how the features were processed may also explain these discrepancies, as well as the inclusion of the group with genetic mutation and the use of a different classifier^6^.

Using the median performance of the Random Forest classifier, we found an operating point where the accuracy reached 81.4% and a true positive rate of 93.8%. The Youden index resulted in the best classification compared with the most commonly used operating point (threshold of 0.5) and with the one obtained with a true positive rate of 95% or 100%, but its false positive rate was 25%. In this study, we considered a preventive approach and prioritized the identification of patients who were about to develop dyskinesia instead of minimizing the rate of falses positives, but this is an important limitation that must be taken into consideration in future studies, especially when testing pharmaceutical or invasive strategies. When analyzing the rules of the decision tree, some features showed to be more important than others for adequately classifying the patients, such as the semantic fluency. Verbal fluency has already been investigated in the context of movement disorders, and it has been demonstrated to change in response to levodopa treatment^20^, with significant differences between the on and off periods in PD patients. In^21^, a significant association between verbal fluency and brain right-sided motor symptoms was demonstrated in PD patients. The possible explanation provided by the authors is that semantic fluency is weighted on language, which is a predominantly left-side cognitive function. We haven’t investigated any association between the most affected side of the body and the development of dyskinesia, but in the study of Eusebi^6^, no significant differences between sides have been found in patients from the PPMI dataset. Future studies are needed to investigate the existence of an association between the side of the body most affected by the PD symptoms and the development of dyskinesia, and consequently if dyskinesia may be specifically associated with the severity of the degenerative process in one of the brain hemispheres.

The UPDRS III score is also a feature that generated a rule able to adequately classify the patients, and this is probably the reason why this feature was regarded as the most important for the Random Forest classifier. The severity of the PD motor symptoms has indeed been considered a risk factor of LID in previous studies^6,22^, and has been demonstrated to be closely related to the levodopa dosage. A total UPDRS score lower than 37.5 appeared as part of the rules considered good to identify patients with higher probability of developing dyskinesia, which was somewhat unexpected. While the literature has shown a positive association between motor symptoms and the development of dyskinesia ^6,22^, the interaction between the severity of non motor symptoms and dyskinesia is less clear. The fact that the total score of the UPDRS represents the severity of both motor and non motor symptoms could explain this result. In our study, the levodopa LEDD also resulted in rules with lift values below one, suggesting that high doses of levodopa may contribute to the development of dyskinesias as demonstrated by Eusebi et al.^6^, Pandey et al.^8^, Olanow et al. 2013, and Dias et al.^23^.

In sum, our study adds to the previous literature by comparing the importance of more than 50 features regarding neurological, clinical and behavioral characteristics in a single sample of patients, and identifying the most important ones for the prediction of dyskinesia. Motor symptoms severity and verbal fluency are individual characteristics of the patients that may be used to identify those at risk of developing dyskinesia. Levodopa dosage is an external and modifiable feature that might be used not only to identify patients at risk of developing dyskinesia, but also be considered in future studies aiming to prevent or delay dyskinesia onset.

## Conclusion

Taken together, our findings suggest that PD patients with lower UPDRS—III and semantic fluency scores, as well as those who have been on dopaminergic medication for a longer period of time, have low risk of developing dyskinesia. On the other hand, patients with higher UPDRS—III and semantic fluency scores, taking levodopa for a shorter time and in higher doses are more likely to develop dyskinesia in the near future. These findings may be considered in future clinical trials aimed at developing therapeutic strategies for the prevention and treatment of levodopa-induced dyskinesia (LID).

### Supplementary Information

Supplementary Information.

## Data Availability

The data that support the findings of this study are available from PPMI (https://www.ppmi-info.org/) but restrictions apply to the availability of these data, which were used under license for the current study, and so are not publicly available. Data are however available from the authors upon reasonable request and with permission of PPMI.

## References

[1] Balestrino R, Schapira AH (2020). Parkinson disease. Eur. J. Neurol..

[2] Marras C (2018). Prevalence of Parkinson's disease across North America. NPJ Parkinson. Dis..

[3] Urso D (2020). Improving the delivery of levodopa in Parkinson’s disease: A review of approved and emerging therapies. CNS Drugs.

[4] Zesiewicz TA, Sullivan KL, Hauser RA (2007). Levodopa-induced dyskinesia in parkinson's disease: Epidemiology, etiology, and treatment. Curr. Neurol. Neurosci. Rep..

[5] Voon V (2017). Impulse control disorders and levodopa-induced dyskinesias in Parkinson's disease: An update. Lancet Neurol..

[6] Eusebi P (2018). Risk factors of levodopa-induced dyskinesia in Parkinson's disease: Results from the ppmi cohort. Npj Parkinson. Dis..

[7] Warren OC (2013). Factors predictive of the development of levodopa-induced dyskinesia and wearing-off in Parkinson's disease. Mov. Disord..

[8] Pandey S, Prachaya S (2017). levodopa-induced dyskinesia: Clinical features, pathophysiology, and medical management. Ann. Indian Acad. Neurol..

[9] Coelho BFO (2023). Parkinson’s disease effective biomarkers based on Hjorth features improved by machine learning. Expert Syst. Appl..

[10] Nancy-Noella RS, Priyadarshini J (2023). Machine learning algorithms for the diagnosis of Alzheimer and Parkinson disease. J. Med. Eng. Technol..

[11] Zhan A (2018). Using smartphones and machine learning to quantify Parkinson disease severity: The mobile Parkinson disease score. JAMA Neurol..

[12] Marek K (2011). The Parkinson's progression marker initiative (ppmi). Progress Neurobiol..

[13] Paquette MA (2012). Anti-dyskinetic mechanisms of amantadine and dextromethorphan in the 6-OHDA rat model of Parkinson’s disease: Role of NMDA vs. 5-HT1A receptors. Eur. J. Neurosci..

[14] Stekhoven DJ, Bühlmann P (2012). Missforest-non-parametric missing value imputation for mixed-type data. Bioinformatics.

[15] Das K, Rabi NB (2017). A survey on machine learning: Concept, algorithms and applications. Int. J. Innov. Res. Comput. Commun. Eng..

[16] Scikit Learn. Decision Trees—scikit-learn 1.0.2 documentation (2021, accessed Dec 2021). https://scikit-learn.org/stable/modules/tree.html#tree.

[17] Huq, S., Tanveer U. l. & Vadlamani R. Evolutionary multi-objective optimization framework for mining association rules. arXiv:2003.09158 (2020).

[18] Ovallath S, Bahiya S (2017). Levodopa: History and therapeutic applications. Ann. Indian Acad. Neurol..

[19] Nicoletti A (2016). Clinical phenotype and risk of levodopa-induced dyskinesia in parkinson's disease. J. Neurol..

[20] Caillava-Santos F, Margis R, De-Mello-Rieder CR (2015). Wearing-off in Parkinson’s disease: Neuropsychological differences between on and off periods. Neuropsychiatr. Dis. Treatment.

[21] Cooper CA (2009). Does laterality of motor impairment tell us something about cognition in Parkinson disease?. Parkinson. Relat. Disord..

[22] Tran TN, Vo TNN, Frei K (2018). Levodopa-induced dyskinesia: Clinical features, incidence, and risk factors. J. Neural Transm..

[23] Dias CMV, Leal DA, Brys I (2022). Levodopa-induced dyskinesia is preceded by increased levels of anxiety and motor impairment in Parkinson's disease patients. Int. J. Neurosci..

