# Supplementary material for: Prediction of dyskinesia in Parkinson’s disease patients using machine learning algorithms
Source: Sci Rep. 2023 Dec 16;13:22426. doi: 10.1038/s41598-023-49617-w (PMC10725420; doi:10.1038/s41598-023-49617-w)
Supplement: Supplementary file 1 — Supplementary Information. [file 41598_2023_49617_MOESM1_ESM.docx]

**Supplementary Material 1**

We employed a randomized grid search for the task, and assessed the performance using the area under the ROC curve. This assessment utilized 10-fold cross-validation with a maximum of 100 sampled configurations. The table below displays the available parameter options for each of the classifiers.

| **Classifier** | **param_grid** |
| --- | --- |
| Adaboost | param_grid = {  'n_estimators': [10, 20, ..., 90],  'learning_rate': [0.1, 0.5, 1, 1.5],  'algorithm': ['SAMME', 'SAMME.R'],  } |
| Decision Tree | param_grid = {  'criterion': ['gini', 'entropy'],  'splitter': ['best', 'random'],  'max_leaf_nodes': [2, 3, 4, ..., 99],  'min_samples_split': [2, 3, 4],  'max_features': [None, 2, 3, 'auto', 'sqrt', 'log2'],  } |
| Logistic Regression | param_grid = {  'solver': ['newton-cg', 'lbfgs', 'liblinear', 'sag', 'saga'],  'max_iter': [50, 100, 150],  'C': [0.001, 0.01, 0.1, 1, 10],  } |
| Multilayer Perceptron | param_grid = {  'activation': ['identity', 'logistic', 'tanh', 'relu'],  'solver': ['lbfgs', 'sgd', 'adam'],  'learning_rate': ['constant', 'invscaling', 'adaptive'],  'hidden_layer_sizes': (  # a layer (X) where X follows the sequence 1, 10, 20, 30, …,  100  # two layers (X, Y) where both vary from 10 to 100 in steps  of 10  ),  } |
| Multinomial | param_grid = {} |
| Random Forest | param_grid = {  'bootstrap': [True],  'max_depth': [80, 90, 100, 110],  'max_features': [2, 3, 'auto', 'sqrt', 'log2'],  'min_samples_leaf': [3, 4, 5],  'min_samples_split': [8, 10, 12],  'n_estimators': [100, 200, 300, 500, 750, 1000],  'criterion': ['gini', 'entropy'],  } |
| Support Vector Machine | param_grid = {  'probability': [True],  'C': [0.001, 0.01, 0.1, 1, 10],  'gamma': [0.001, 0.01, 0.1, 1],  'kernel': ['rbf', 'poly', 'sigmoid'],  } |
